# The pathogen recognition sensor, NOD2, is variably expressed in patients with pulmonary tuberculosis

**DOI:** 10.1186/1471-2334-7-96

**Published:** 2007-08-16

**Authors:** Sanjay Lala, Keertan Dheda, Jung-Su Chang, Jim F Huggett, Louise U Kim, Margaret A Johnson, Graham AW Rook, Satish Keshav, Alimuddin Zumla

**Affiliations:** 1 Centre for Gastroenterology, Royal Free and University College Medical School & Royal Free Hospital NHS Trust, London, UK; 2 Centre for Infectious Diseases and International Health, Royal Free Hospital NHS Trust, London, UK; 3 Lung Infection and Immunity Unit, Department of Medicine, Division of Pulmonology, University of Cape Town, South Africa; 4 Dept. of Thoracic and HIV Medicine, Royal Free Hospital NHS Trust, London, UK

## Abstract

**Background:**

NOD2, an intracellular pathogen recognition sensor, modulates innate defences to muropeptides derived from various bacterial species, including *Mycobacterium tuberculosis *(MTB). Experimentally, NOD2 attenuates two key putative mycobactericidal mechanisms. TNF-α synthesis is markedly reduced in MTB-antigen stimulated-mononuclear cells expressing mutant NOD2 proteins. NOD2 agonists also induce resistance to apoptosis, and may thus facilitate the survival of MTB in infected macrophages. To further define a role for NOD2 in disease pathogenesis, we analysed NOD2 transcriptional responses in pulmonary leucocytes and mononuclear cells harvested from patients with pulmonary tuberculosis (PTB).

**Methods:**

We analysed NOD2 mRNA expression by real-time polymerase chain-reaction in alveolar lavage cells obtained from 15 patients with pulmonary tuberculosis and their matched controls. We compared NOD2 transcriptional responses, in peripheral leucocytes, before and after anti-tuberculous treatment in 10 patients. *In vitro*, we measured NOD2 mRNA levels in MTB-antigen stimulated-mononuclear cells.

**Results:**

No significant differences in NOD2 transcriptional responses were detected in patients and controls. In some patients, however, NOD2 expression was markedly increased and correlated with toll-like-receptor 2 and 4 expression. In whole blood, NOD2 mRNA levels increased significantly after completion of anti-tuberculosis treatment. NOD2 expression levels did not change significantly in mononuclear cells stimulated with mycobacterial antigens *in vitro*.

**Conclusion:**

There are no characteristic NOD2 transcriptional responses in PTB. Nonetheless, the increased levels of NOD2 expression in some patients with severe tuberculosis, and the increases in expression levels within peripheral leucocytes following treatment merit further studies in selected patient and control populations.

## Background

The innate immune system is postulated to play a crucial role in the elimination or control of *Mycobacterium tuberculosis *(MTB), which causes infectious tuberculosis [1,2]. These innate immune responses are triggered when MTB-derived molecules are sensed by toll-like receptors (TLRs), a family of membrane proteins, as well as by nucleotide-binding oligomerization domain 2 (NOD2; also known as CARD15), an intracellular pathogen recognition sensor. Activation of TLRs by MTB-antigens appears to be an important event in the control of infection. For example, mice that lack the toll-like receptor (TLR) adaptor molecule myeloid differentiation factor 88 (MyD88) are more susceptible to pneumonia following aerogenic infection with MTB [3].

The nature of these innate responses, however, appears complex and is not fully elucidated. For example, experimental studies show that MTB-induced TLR signalling effects both cellular activation [4] and apoptosis [5]. Similarly, the innate responses mediated by NOD2, in response to MTB-derived antigens, vary in different experimental systems.

NOD2 senses muramyl dipeptide (MDP) [6,7] a component of peptigoglycan that is found in mycobacterial cell walls. A recent *in vitro *study suggests that NOD2 is an essential recognition molecule for MTB [8], and pronounced cellular activation is noted in NOD2-transfected cells that are stimulated with MDP or heat-killed MTB preparations. Furthermore, cytokine production is inhibited in MTB-stimulated peritoneal macrophages obtained from NOD2-deficient mice and in human peripheral blood mononuclear cells (PBMCs) that express truncated NOD2 proteins [8]. On the other hand, MDP stimulation protects macrophages from apoptosis, which suggests that activation of NOD2 induces apoptosis resistance which facilitates the survival of MTB in macrophages [5].

The role of NOD2 in the pathogenesis of human MTB-infection is unknown. *In vitro*, human PBMCs that express truncated NOD2 proteins synthesise significantly less cytokines after stimulation with MTB-derived antigens. Truncated NOD2 proteins are encoded by mutations in the NOD2 gene that predispose individuals to Crohn's disease, a granulomatous inflammatory bowel disease [9,10]. The prevalence of these Crohn's disease-associated mutations has not been fully described in all population groups where tuberculosis is endemic although these mutations are rare in African patients with tuberculosis [11].

To further define a role for NOD2 in disease pathogenesis, we analysed NOD2 mRNA transcriptional responses in pulmonary leucocytes and PBMCs harvested from patients with pulmonary tuberculosis (PTB) and healthy controls. We determined whether changes in NOD2 transcription, if present, are characteristic for patients with tuberculosis: increased transcriptional responses may suggest that MTB-infected macrophages are resistant to apoptosis whereas decreased transcriptional responses may suggest that cellular activation is diminished in infected macrophages. As various pathogen associated-molecular patterns (PAMPs) specifically alter host transcriptional responses [12], we analysed and correlated the transcriptional responses of other TLRs with NOD2 in patients with PTB.

## Methods

### Patients and samples

Fifteen HIV-negative patients with culture proven pulmonary tuberculosis (PTB), who donated blood and lung bronchoalveolar lavage (BAL) samples, were recruited in London, United Kingdom. All patients with PTB had pan-sensitive isolates, received standard short course chemotherapy (6 to 9 months) and demonstrated clinical and radiological response to anti-TB treatment. Control donors (n = 15) were healthy volunteers matched to the TB patients for age (within 4 years), sex and ethnicity. They were asymptomatic, had no risk factors for HIV infection (but were not formally tested), had normal chest radiographs and were assumed, based on antigen-specific (ESAT-6 and CFP-10) peripheral mononuclear cell IFN-γ responses, not to be latently infected (T SPOT TB, Oxford Immunotec, England) [13]. Control BAL samples were obtained from six control donors.

Whole blood (20 mL) was taken, after informed consent, within the first 2 weeks of anti-TB treatment (baseline). 2.5 mL whole blood was immediately transferred into PAXgene Blood RNA Tubes (Qiagen) for isolation and purification of intracellular RNA. The remaining blood, where relevant, was used for further experiments. Ten donors with PTB were bled again within 4 weeks of stopping chemotherapy. Approval was obtained from the Royal Free and UCLH hospital ethics committees.

### BAL and Radiographic scoring

BAL fluid, obtained from a radiologically affected lung segment, was concentrated ~10 fold before analysis whilst cell pellets were immediately fixed in RNA stabilisation buffer. In control donors the right middle lobe was lavaged. When possible, lymphocyte counts in BAL were confirmed by flow cytometry (10^4 ^gated events) after staining ~7.5 × 10^5 ^cells with anti CD4-FITC, anti CD8-PE and anti CD3-PercP antibodies (BD Biosciences, UK). To determine the extent of pre-treatment radiological disease, two radiologists, blinded to patient details, scored chest radiographs for air space shadowing, reticular opacities and cavitation.

### Enzyme-linked-immunospot (ELISPOT) assays

Peripheral T-cell IFN-γ ELISPOT responses to ESAT-6 and CFP-10 peptide pools were determined to exclude latent TB infection (T SPOT TB, Oxford Immunotec, England), as previously described [14,13,15].

### Culture and infection of PBMCs with *M. tuberculosis*

PBMCs from five healthy control donors were separated from heparinized blood (50 ml) by Ficoll density gradient centrifugation, and cells were reconstituted at a final concentration of 1 × 10^6 ^cells/ml. To evaluate NOD2 gene regulation PBMCs were cultured for 66 hours in the presence of live H37RV *M. tuberculosis*, a clinical isolate (Beijing strain), live environmental mycobacterium (*M. vaccae *NCTC 11659) and medium alone. Cells were cultured in RPMI 1640 supplemented with 5% heat-inactivated human AB serum and 1% L-glutamine without antibiotics at 37°C and 5% CO_2 _in a category 3 laboratory and harvested at 18, 24, 48, and 66 h post-treatment. All strains were grown in Middlebrook 7H10 agar (Difco) containing 10% v/v oleic acid/albumin/dextrose/catalase supplement (BD Biosciences) in the category 3 laboratory. Mycobacteria were disaggregated by vigorous vortexing with glass beads and counted using a Neubauer hemocytometer as previously described [16]. A direct microscopic count was performed to determine mycobacterial concentration. PBMCs were infected with living mycobacteria at a dose of 1 organism per macrophage (10% monocytes in PBMC). The viability of mycobacteria was assessed by culturing the diluted bacteria on Middlebrook 7H10 agar.

### Reverse transcription and real-time PCR

RNA was isolated from whole blood and from lavage cell pellets or cells using the Paxgene^® ^and RNeasy^® ^Kit, respectively. Reverse transcription and real-time PCR, to quantify mRNA encoding for NOD2 and NOD1, IL-4 and its splice variant IL-4δ2, IFN-γ, TLRs and several proteins involved in apoptosis (FLIP, FLICE, Bcl-2, Bax, Fas, FasL and Bfl-1), were performed on samples, as previously described, after quality control of RNA templates [17]. mRNA values were normalised to a validated housekeeping gene, human-acidic-ribosomal-protein (HuPO) [18,19]. Primer and probe sequences, excluding those which have been previously published [16,17], are shown in table 2.

**Table 2:** Primer and probe sequences used to quantify gene expression by real-time PCR. Primer sequences for toll-like-receptor 2, 4, 6, 7 and 9 [17] and IL-4δ2 [18] have previously been published.

Genes	* Probe sequence 5'-(FAM-TAMRA)-3' ◇ L primer-5'-3' □ R primer-5'-3'	Product size
IL-4	* AAACCTTCTGCAGGGCTGCGAC	71 bp
	◇ GCTGCCTCCAAGAACACAAC	
	□ CTGTAGAACTGCCGGAGCAC	

HuPO	*TGCCAGTGTCTGTCTGCAGATTGG	105 bp
	◇ GCTTCCTGGAGGGTGTCC	
	□ GGACTCGTTTGTACCCGTTG	

NOD1	* CCTGGCTCCGACATCGGTGA	133 bp
	◇ AAGCGAAGAGCTGACCAAAT	
	□ TCCCAGTTTAAGATGCGTGA	

NOD2	* CCGAGGCATCTGCAAGCTCA	82 bp
	◇ CTGCAAGGCTCTGTATTTGC	
	□ CTCGCAGTGAAGAGCACATT	

TNF-α	* CAGCCACTGGAGCTGCCCCT	102 bp
	◇ AGCCCATGTTGTAGCAAACC	
	□ GCTGGTTATCTCTCAGCTCCA	

FLIP	* TGGATTGCTGCTTGGAGAACATTCC	114 bp
	◇ GTTCAAGGAGCAGGGACAAG	
	□ ATCAGGACAATGGGCATAGG	

FLICE	* ACTTGGATGCAGGGGCTTTGACCAC	102 bp
	◇ AAGTGCCCAAACTTCACAGC	
	□ GGGGCTTGATCTCAAAATGA	

Bax	* AAGTAGAAAAGGGCGACAACCCGGC	105 bp
	◇ GAGAGGTCTTTTTCCGAGTGG	
	□ GCCTTGAGCACCAGTTTGCTG	

Bfl-1	* CCACAACCTGGATCAGGTCCAAGCA	105 bp
	◇ GGCTGGCTCAGGACTATCTG	
	□ TTTGGACTGAGAACGCAACA	

Bcl-2	* CAAAGGCATCCCAGCCTCCGTTA	118 bp
	◇ AGTACCTGAACCGGCACCT	
	□ TTCAGAGACAGCCAGGAGAAA	

### Data analysis

Data was analysed using the Mann-Whitney U test, Wilcoxon matched pairs test, Spearman rank-sum correlation and linear regression on logged data.

## Results

### Demographic characteristics

Demographic details of matched TB patients (n = 15) and controls, shown in Table 1, indicate that there were no differences in baseline characteristics between these groups. All control patients had normal chest radiographs and had no laboratory evidence of latent TB infection with *M. tuberculosis*, as assessed by the antigen specific IFN-γ assay (T SPOT TB).

**Table 1:** Demographic details and lavage cell counts at recruitment. Demographic details and radiographic scores are of 15 patients with pulmonary tuberculosis with their matched controls; the lavage cell counts are those of 10 patients and 6 controls. All participants were recruited in London, United Kingdom.

Numbers in parenthesis indicate %	TB	Control
Age (median; range in years)	28; 18–52	28; 19–48
Sex (male)	9 (60)	8 (53)

Ethnicity		
Black African	9 (60)	8 (53)
Indian	4 (26)	4 (26)
Other	2 (14)	3 (21)

X-ray features		
Cavitation	8 (53)	0
>50% airspace shadowing of total lung field	7 (47)	0

**BAL (mean ± SE)**		
Total leucocytes (× 10^4^/ml)	31 ± 8	12.8 ± 4.2
lymphocyte count (× 10^4^/ml)	15 ± 4	2.7 ± 1.4
CD4: CD8 ratio	3.8 ± 1	2.55 ± 0.9
IFN-γ copy number	3390 [1525–7498]	13 [4–113]; p = 0.0005
Th1/Th2 ratio	3.5 [3.1–3.8]	1.1 [.4–1.8]; p = 0.0007

### Broncho-alveolar lavage (BAL) fluid analyses in patients with PTB and controls

BAL fluid recovered from patients with PTB were more cellular than controls, contained predominantly alveolar macrophages, and contained a significantly greater number of leucocytes and lymphocytes (Table 1). In PTB-affected patients, IFN-γ mRNA levels and the Th1/Th2 (IFN-γ/IL-4) ratio were significantly elevated compared to control subjects (Table 1).

### Pulmonary leucocytes express NOD2 mRNA

**Figure 1:**
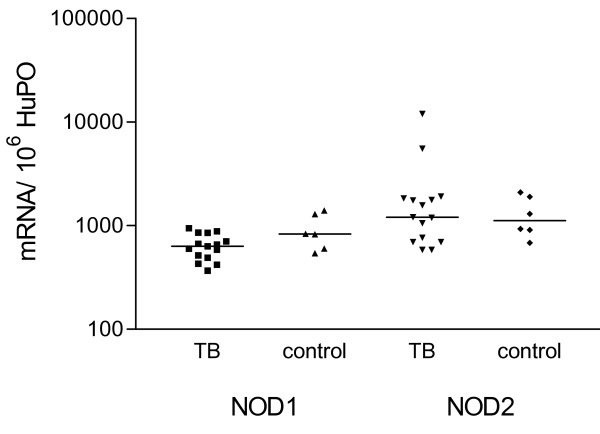
**NOD1 and NOD2 mRNA expression in pulmonary leucocytes obtained from patients with tuberculosis and controls**. NOD1 and NOD2 mRNA expression, measured by real-time RT-PCR, is similar in pulmonary leucocytes in 15 patients with tuberculosis compared to 6 healthy controls. To permit comparison between individuals, absolute copy numbers of NOD1 and NOD2 mRNA were measured during RT-PCR, using cDNA standards, and expressed relative to 10^6 ^mRNA copies of a validated housekeeping gene, HuPO.

NOD2 is most prominently expressed in circulating monocytes [10] and small intestinal Paneth cells [20]. Expression profiles in human pulmonary leucocytes have not previously been studied. We initially determined whether alveolar macrophages express NOD2 and measured NOD2 mRNA levels in pulmonary leucocytes present in BAL fluid [21]. We show that NOD2 mRNA expression levels are prominent in pulmonary leucocytes although expression levels did not differ between patients and controls (Figure 1). We also compared the expression levels of NOD1 mRNA (also known as CARD4), in pulmonary leucocytes obtained from patients and controls (Figure 1). NOD1 is an intracellular protein that is closely related to NOD2 but does not appear to play a role in the detection of mycobacterial antigens [8].

**Figure 2:**
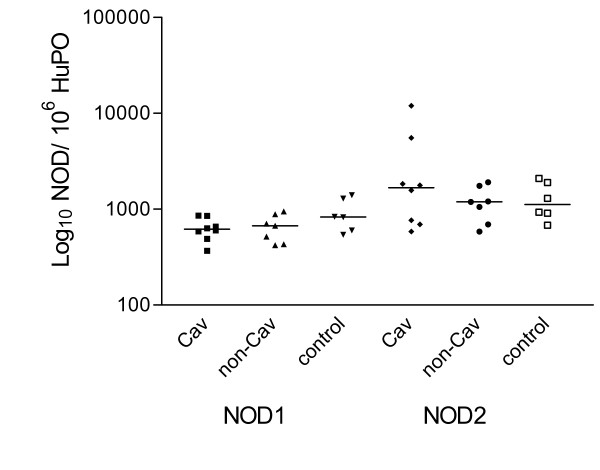
NOD2 gene expression in alveolar lavage cells from 15 subjects with tuberculosis (stratified by whether cavitation was detected on chest radiographs) and 6 healthy controls.

Although there are no characteristic NOD2 transcriptional responses in patients with PTB, NOD2 mRNA was highly expressed in two patients. Both patients, who were of Somalian origin, and had severe tuberculosis: one patient had extensive pulmonary cavitatory disease and the other disseminated tuberculosis. We could not, however, identify more specific clinical variables to account for the high levels of NOD2 expression in these patients. Patients with cavitatory disease tended to have higher levels of NOD2 mRNA expression than those without pulmonary cavitation but these differences were not significant (Figure 2). There was no correlation between NOD2 mRNA levels and radiographic disease scores.

### NOD2, and TLR2 and TLR4 mRNA expression correlate in pulmonary leucocytes obtained from patients with PTB

**Figure 3:**
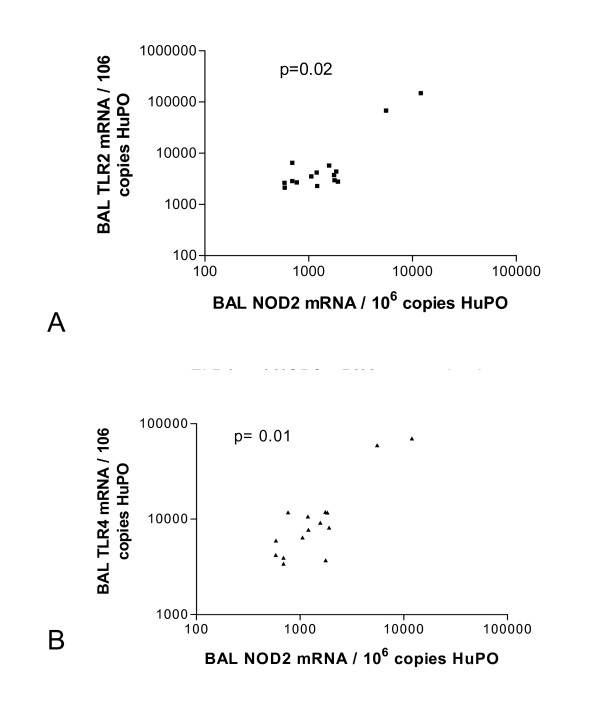
**NOD2 mRNA expression correlates with TLR2 and TLR4 mRNA expression in pulmonary leucocytes harvested from patients with tuberculosis**. A significant correlation was observed between mRNA molecules encoding NOD2 and toll-like receptor (TLR) 2 (p = 0.02) [A] and TLR4 (p = 0.01) [B] in pulmonary leucocytes harvested from patients with tuberculosis. To permit correlations, absolute copy numbers of NOD2 and TLR2, TLR4 and TLR6 mRNA were measured during RT-PCR, using cDNA standards, and expressed relative to 10^6 ^mRNA copies of a validated housekeeping gene HuPO.

NOD2 regulates cellular responses to peptidoglycan-mediated activation of TLR2 [22]. Furthermore, mycobacterial TLR2 and NOD2 agonists synergistically induce cytokine release in PBMCs obtained from healthy volunteers and patients with Crohn's disease [8]. In pulmonary leucocytes obtained from patients with tuberculosis, NOD2 mRNA expression correlated with TLR2 (p = 0.02; figure 3A) and TLR4 (p = 0.01; figure 3B) but not with TLR6 mRNA expression (p = 0.17). These correlations were mainly due to the high levels of mRNA expression in the two previously mentioned patients. In healthy controls, there was no correlation between NOD2 and TLR2 or TLR4 mRNA expression in pulmonary leucocytes. NOD1 mRNA expression did not correlate with TLR2 or TLR4 mRNA expression in pulmonary leucocytes obtained from patients and controls.

### NOD2 expression increases in whole blood following anti-tuberculous therapy

**Figure 4:**
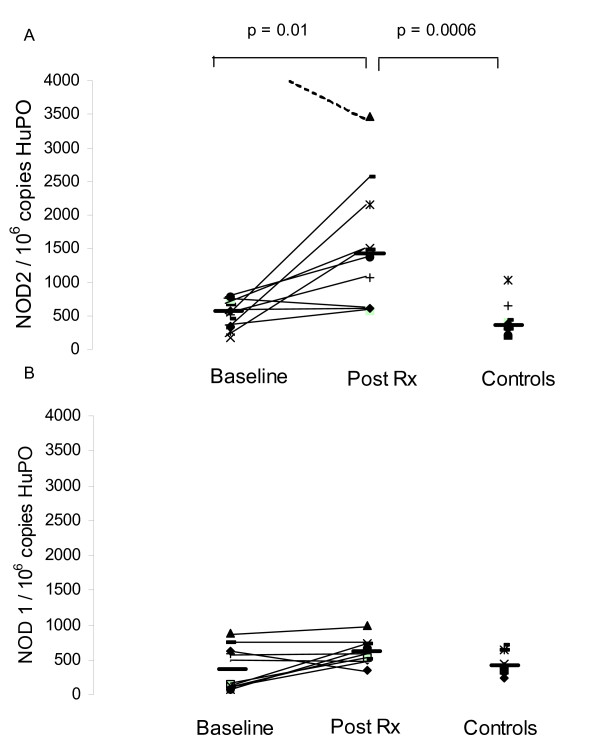
**NOD1 and NOD2 mRNA expression in peripheral blood cells obtained from patients with tuberculosis, before and after anti-tuberculous treatment**. NOD1 and NOD2 mRNA expression was analysed, by real-time RT-PCR, in peripheral blood cells isolated from patients with tuberculosis and matched controls. To permit comparison between individuals, absolute copy numbers of NOD2 (4A) and NOD1 (4B) mRNA were measured during RT-PCR, using cDNA standards, and expressed relative to 10^6 ^mRNA copies of a validated housekeeping gene HuPO. NOD2 but not NOD1 mRNA levels increased significantly after treatment.

In whole blood, both NOD2 and NOD1 mRNA expression increased following treatment, although only increases in NOD2 mRNA expression were significant (Figure 4).

### Signalling pathways activated by NOD2

Following activation by MDP, NOD2 triggers downstream cellular responses mediated through NF-κB and mitogen-activated protein kinase (MAPK) activation [23]. This results in the production of various cytokines, such as TNFα and IL-4, which are important in regulating immune responses to MTB [1,17]. We therefore compared NOD2 mRNA with the transcriptional responses of selected cytokines in BAL-derived cells obtained from patients with PTB (n = 15) and controls (n = 6). Although there were clear differences in the expression of some cytokines (IFNγ, IL-4 and its splice variant and antagonist, IL-4δ2) in patients with TB and controls, there was no correlation between expression of these cytokines and NOD2. There was also no correlation between NOD2 and TNFα transcriptional responses in patients and controls. In addition, NOD2 mRNA expression did not correlate with expression of mRNA encoding several proteins involved in apoptosis (FLIP, FLICE, Bcl-2, Bax, Fas, FasL and Bfl-1) although high levels of mRNA encoding the anti-apoptotic protein FLICE were measured in BAL-derived cells obtained from the two patients with elevated NOD2 mRNA levels.

### NOD2 mRNA expression is not regulated in PMBC freshly inoculated with live strains of mycobacteria

**Figure 5:**
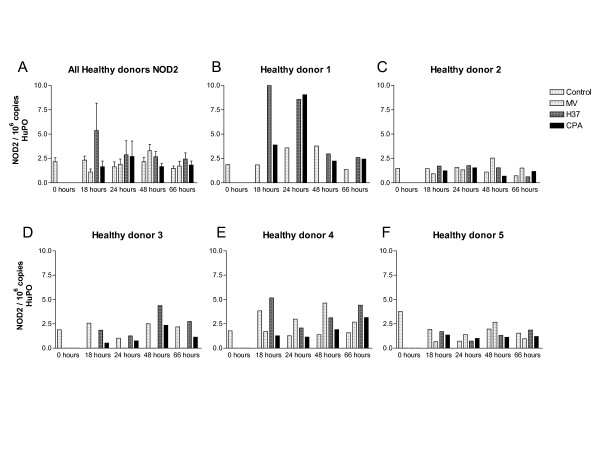
**NOD2 mRNA expression in live mycobacteria stimulated-peripheral blood cells obtained from healthy volunteers**. NOD2 mRNA levels varied widely in healthy donor derived-PBMCs that were stimulated with live mycobacterial strains [A = combined data and B to F individual data]. NOD2 mRNA expression was analysed, by real-time RT-PCR, in peripheral blood cells isolated from healthy donors. To permit comparison between individuals, absolute copy numbers of NOD2 mRNA were measured during RT-PCR, using cDNA standards, and expressed relative to 10^6 ^mRNA copies of a validated housekeeping gene HuPO. Lower numbers of PBMCs were harvested from Donors 1 and 3, and these cells were not stimulated with *Mycobacterium vaccae*. MV = M, vaccae; H37 = H37RV strain and CPA = Beijing strain.

We exposed PBMCs from five healthy volunteers, who did not have laboratory evidence of tuberculosis infection (as established by antigen-specific IFN-γ assays [14,13,15]), to pathogenic strains of live mycobacteria (H37RV and Beijing strain) and an environmental mycobacterium (*M. vaccae *NCTC 11659), to determine if NOD2 mRNA expression levels are regulated by. In general, mycobacteria induced modest, but non-significant changes, in NOD2 mRNA expression (Figure 5A). Mycobacteria induced marked increases in NOD2 mRNA expression in one volunteer (Figure 5B) although mycobacterial stimulation did not regulate NOD2 mRNA expression significantly in PBMC extracted from the other volunteers (Figure 5C–F). In these studies, NOD2 mRNA expression levels varied widely and did not correlate with any specific mycobacterial strain.

## Discussion

NOD2, a member of the phylogenetically conserved NLR (NACHT-leucine-rich repeat) family, is an essential pattern recognition sensor for MTB-derived antigens. Mycobacterial antigens fail to induce an appropriate increase in TNF-α synthesis in human PBMC that express mutant NOD2 proteins and in murine macrophages lacking NOD2 [8]. The gene mutations that encode mutant NOD2 proteins, however, appear to be rare in patients with tuberculosis. NOD2 agonists may also modulate innate responses to MTB by inducing resistance to apoptosis that facilitates the survival of MTB in infected macrophages [5]. Thus, NOD2 may play a role in attenuating two key putative mycobactericidal pathways. To further define a role for NOD2 in disease pathogenesis, we analysed NOD2 transcriptional responses in pulmonary leucocytes and mononuclear cells harvested from patients with pulmonary tuberculosis (PTB).

Our gene expression studies revealed that there are no characteristic NOD2 transcriptional responses in pulmonary leucocytes obtained from patients with tuberculosis. NOD2 mRNA levels in patients generally compared with those in control donors. Nonetheless, increased NOD2 levels, which correlate with TLR2 and TLR4 expression, were noted in some patients with severe infection. This observation, coupled with the increases in NOD2 expression in peripheral leucocytes following treatment, suggest further study in a larger group of patients to confirm a role for NOD2 in PTB. In the present study, we only measured total leucocyte and lymphocyte counts in bronchoalveolar lavage fluid. As NOD2 is most prominently expressed in monocytes, with very little expression in neutrophils and lymphocytes, it is most likely that monocytes account for the overwhelming majority of NOD2 expression [24].

We did not quantify the number of epithelial cells present in broncho-alveolar fluid and we cannot determine the effect of mycobacterial infection on NOD2 expression in respiratory epithelial cells. Baseline NOD2 expression levels in primary respiratory epithelial cells is low [25] although NOD2 mRNA expression is enhanced in immortalised human bronchial epithelial cells that are infected with *Streptococcus pneumoniae*[25]. It would therefore be important to determine the relative contribution of respiratory epithelial cells and monocytes to the increases in NOD2 expression seen in some patients.

In the present study, NOD2 mRNA expression levels were similar in patients (who often have active disease for many weeks prior to diagnosis) and controls. This does not exclude a role for NOD2 during the early stages of MTB infection, when *M. tuberculosis *encounters the alveolar macrophage and innate immune pathways are first activated. It is also possible, however, that the absolute levels of NOD2 expression may not play a role in determining susceptibility to MTB infection. Rather, structural variants of NOD2 proteins may modulate host immune responses as suggested by *in vitro *studies [8]. Thus, studies are required to confirm whether individuals with gene mutations encoding for mutant NOD2 proteins are predisposed to MTB-infection. The lack of an association between Crohn's disease-associated NOD2 gene mutations and tuberculosis in African patients [11] does not exclude a role for NOD2 in MTB-infection because NOD2 gene mutations are probably rare in African populations [10]. It would be instructive, therefore, to determine whether NOD2 gene mutations are associated with MTB-infection in Caucasian populations, where these mutations occur with much greater frequency.

We were surprised to find significantly higher levels of NOD2 mRNA expression in peripheral leucocytes obtained from patients who completed anti-tuberculosis therapy. Firstly, we hypothesise that this could have been due to translocation of antigen-specific leucocytes predominantly to the site of disease (lungs) with few NOD2 expressing leucocytes in the peripheral compartment, and reversal of this profile after treatment. However, the lack of preferential NOD2 upregulation in the lung makes this unlikely. Secondly, we speculate that MTB infection may subvert protective innate responses by downregulating NOD2 expression, whose levels therefore increase after successful chemotherapy. We might expect this to occur in parallel with TNF-α as this cytokine up-regulates NOD2 mRNA expression in various cell lineages including PBMC [26,27]. However, in keeping with the observations of other investigators [28,29], we did not observe increased TNF-α mRNA expression after treatment completion. We did not investigate the relationship between NOD2 and soluble TNF-α receptors, which may modulate TNF-α levels. Thirdly, it is intriguing to speculate that increased levels of NOD2 mRNA, which occur with completion of TB treatment, is a correlate of protective immunity. Similar longitudinal changes may occur with IFN-γ [17,29,30] and the Th1-like splice variant IL-4δ2 [17], which both increase significantly with anti-TB treatment. In keeping with these observations IL-4δ2 mRNA levels are also increased in healthy subjects with latent MTB infection who contain the disease [31,32]. Longitudinal studies in TB infected patients, however, would be required to address the role of NOD2 in this context. Interestingly, preliminary data indicate that mycobacterial antigens regulate the expression of NOD2 splice variants [33] and further studies are required to clarify their role in tuberculosis.

A significant limitation of this study is that we did not study MTB-specific responses in subjects with known NOD2 gene mutations. However, we found these patients difficult to recruit in our clinical setting. We also acknowledge that real time PCR measures steady state mRNA levels only and not the activity of NOD2 protein, which is physiologically active at concentrations too low to detect by immunoassay. This study was powered to detect a 0.5 log change in NOD2 mRNA levels although smaller changes may be biologically meaningful. However, mRNA levels in patients and controls were similar and we found no trends suggesting that inter-group difference might be present.

## Conclusion

Overall, our findings show that there are no characteristic NOD2 transcriptional responses in pulmonary leucocytes obtained from patients with PTB. Nonetheless, the increased levels of NOD2 mRNA expression in peripheral leucocytes obtained from patients completing treatment and correlation between NOD2 and TLR2 and TLR4 mRNA expression in pulmonary leucocytes obtained from some patients with severe tuberculosis does not exclude a role for NOD2 in disease pathogenesis. The role of mutant NOD2 proteins in MTB-infection in different ethnic groups needs to be defined.

## Competing interests

The author(s) declare that they have no competing interests.

## Authors' contributions

SL contributed to formulation of study design, conducted some of the relevant experiments, analysed the data, and contributed to writing and critical appraisal of the manuscript. KD contributed to formulation of study design, conducted some of the relevant experiments, recruited the patients, analysed the data, and contributed to writing and critical appraisal of the manuscript. JC conducted some of the relevant experiments and contributed to data analysis. LUK conducted some of the relevant experiments and contributed to data analysis. JFH conducted some of the relevant experiments and contributed to data analysis. MAJ, SK, GAWR and AZ contributed to formulation of study design, facilitated patient recruitment, analysed the data, and contributed to writing and critical appraisal of the manuscript. All the authors have read and approved the final manuscript.

## Pre-publication history

The pre-publication history for this paper can be accessed here:



## References

[B1] Dheda K, Booth H, Huggett JF, Johnson MA, Zumla A, Rook GA (2005). Lung remodeling in pulmonary tuberculosis. J Infect Dis.

[B2] North RJ, Jung YJ (2004). Immunity to tuberculosis. Annu Rev Immunol.

[B3] Fremond CM, Yeremeev V, Nicolle DM, Jacobs M, Quesniaux VF, Ryffel B (2004). Fatal Mycobacterium tuberculosis infection despite adaptive immune response in the absence of MyD88. J Clin Invest.

[B4] Doherty TM, Arditi M (2004). TB, or not TB: that is the question -- does TLR signaling hold the answer?. J Clin Invest.

[B5] Loeuillet C, Martinon F, Perez C, Munoz M, Thome M, Meylan PR (2006). Mycobacterium tuberculosis subverts innate immunity to evade specific effectors. J Immunol.

[B6] Girardin SE, Boneca IG, Viala J, Chamaillard M, Labigne A, Thomas G, Philpott DJ, Sansonetti PJ (2003). Nod2 is a general sensor of peptidoglycan through muramyl dipeptide (MDP) detection. J Biol Chem.

[B7] Inohara N, Ogura Y, Fontalba A, Gutierrez O, Pons F, Crespo J, Fukase K, Inamura S, Kusumoto S, Hashimoto M, Foster SJ, Moran AP, Fernandez-Luna JL, Nunez G (2003). Host recognition of bacterial muramyl dipeptide mediated through NOD2. Implications for Crohn's disease. J Biol Chem.

[B8] Ferwerda G, Girardin SE, Kullberg BJ, Le Bourhis L, de Jong DJ, Langenberg DM, van Crevel R, Adema GJ, Ottenhoff TH, Van der Meer JW, Netea MG (2005). NOD2 and toll-like receptors are nonredundant recognition systems of Mycobacterium tuberculosis. PLoS Pathog.

[B9] Hugot JP, Chamaillard M, Zouali H, Lesage S, Cezard JP, Belaiche J, Almer S, Tysk C, O'Morain CA, Gassull M, Binder V, Finkel Y, Cortot A, Modigliani R, Laurent-Puig P, Gower-Rousseau C, Macry J, Colombel JF, Sahbatou M, Thomas G (2001). Association of NOD2 leucine-rich repeat variants with susceptibility to Crohn's disease. Nature.

[B10] Ogura Y, Bonen DK, Inohara N, Nicolae DL, Chen FF, Ramos R, Britton H, Moran T, Karaliuskas R, Duerr RH, Achkar JP, Brant SR, Bayless TM, Kirschner BS, Hanauer SB, Nunez G, Cho JH (2001). A frameshift mutation in NOD2 associated with susceptibility to Crohn's disease. Nature.

[B11] Stockton JC, Howson JM, Awomoyi AA, McAdam KP, Blackwell JM, Newport MJ (2004). Polymorphism in NOD2, Crohn's disease, and susceptibility to pulmonary tuberculosis. FEMS Immunol Med Microbiol.

[B12] Zarember KA, Godowski PJ (2002). Tissue expression of human Toll-like receptors and differential regulation of Toll-like receptor mRNAs in leukocytes in response to microbes, their products, and cytokines. J Immunol.

[B13] Dheda K, Udwadia ZF, Huggett JF, Johnson MA, Rook GA (2005). Utility of the antigen-specific interferon-gamma assay for the management of tuberculosis. Curr Opin Pulm Med.

[B14] Dheda K, Lalvani A, Miller RF, Scott G, Booth H, Johnson MA, Zumla A, Rook GA (2005). Performance of a T-cell-based diagnostic test for tuberculosis infection in HIV-infected individuals is independent of CD4 cell count. Aids.

[B15] Ewer K, Deeks J, Alvarez L, Bryant G, Waller S, Andersen P, Monk P, Lalvani A (2003). Comparison of T-cell-based assay with tuberculin skin test for diagnosis of Mycobacterium tuberculosis infection in a school tuberculosis outbreak. Lancet.

[B16] Chang JS, Huggett JF, Dheda K, Kim LU, Zumla A, Rook GA (2006). Mycobacterium tuberculosis induces selective upregulation of TLRs in the mononuclear leukocytes of patients with active pulmonary tuberculosis. J Immunol.

[B17] Dheda K, Chang JS, Breen RA, Kim LU, Haddock JA, Huggett JF, Johnson MA, Rook GA, Zumla A (2005). In vivo and in vitro studies of a novel cytokine, interleukin 4delta2, in pulmonary tuberculosis. Am J Respir Crit Care Med.

[B18] Dheda K, Huggett JF, Bustin SA, Johnson MA, Rook G, Zumla A (2004). Validation of housekeeping genes for normalizing RNA expression in real-time PCR. Biotechniques.

[B19] Dheda K, Huggett JF, Chang JS, Kim LU, Bustin SA, Johnson MA, Rook GA, Zumla A (2005). The implications of using an inappropriate reference gene for real-time reverse transcription PCR data normalization. Anal Biochem.

[B20] Lala S, Ogura Y, Osborne C, Hor SY, Bromfield A, Davies S, Ogunbiyi O, Nunez G, Keshav S (2003). Crohn's disease and the NOD2 gene: a role for paneth cells. Gastroenterology.

[B21] Mazzarella G, Bianco A, Perna F, D'Auria D, Grella E, Moscariello E, Sanduzzi A (2003). T lymphocyte phenotypic profile in lung segments affected by cavitary and non-cavitary tuberculosis. Clin Exp Immunol.

[B22] Watanabe T, Kitani A, Murray PJ, Strober W (2004). NOD2 is a negative regulator of Toll-like receptor 2-mediated T helper type 1 responses. Nat Immunol.

[B23] Strober W, Murray PJ, Kitani A, Watanabe T (2006). Signalling pathways and molecular interactions of NOD1 and NOD2. Nat Rev Immunol.

[B24] Ogura Y, Inohara N, Benito A, Chen FF, Yamaoka S, Nunez G (2001). Nod2, a Nod1/Apaf-1 family member that is restricted to monocytes and activates NF-kappaB. J Biol Chem.

[B25] Opitz B, Puschel A, Schmeck B, Hocke AC, Rosseau S, Hammerschmidt S, Schumann RR, Suttorp N, Hippenstiel S (2004). Nucleotide-binding oligomerization domain proteins are innate immune receptors for internalized Streptococcus pneumoniae. J Biol Chem.

[B26] Gutierrez O, Pipaon C, Inohara N, Fontalba A, Ogura Y, Prosper F, Nunez G, Fernandez-Luna JL (2002). Induction of Nod2 in myelomonocytic and intestinal epithelial cells via nuclear factor-kappa B activation. J Biol Chem.

[B27] Rosenstiel P, Fantini M, Brautigam K, Kuhbacher T, Waetzig GH, Seegert D, Schreiber S (2003). TNF-alpha and IFN-gamma regulate the expression of the NOD2 (CARD15) gene in human intestinal epithelial cells. Gastroenterology.

[B28] Kart L, Buyukoglan H, Tekin IO, Altin R, Senturk Z, Gulmez I, Demir R, Ozesmi M (2003). Correlation of serum tumor necrosis factor-alpha, interleukin-4 and soluble interleukin-2 receptor levels with radiologic and clinical manifestations in active pulmonary tuberculosis. Mediators Inflamm.

[B29] Ribeiro-Rodrigues R, Resende CT, Johnson JL, Ribeiro F, Palaci M, Sa RT, Maciel EL, Pereira Lima FE, Dettoni V, Toossi Z, Boom WH, Dietze R, Ellner JJ, Hirsch CS (2002). Sputum cytokine levels in patients with pulmonary tuberculosis as early markers of mycobacterial clearance. Clin Diagn Lab Immunol.

[B30] Al Attiyah R, Mustafa AS, Abal AT, Madi NM, Andersen P (2003). Restoration of mycobacterial antigen-induced proliferation and interferon-gamma responses in peripheral blood mononuclear cells of tuberculosis patients upon effective chemotherapy. FEMS ImmunolMedMicrobiol.

[B31] Demissie A, Abebe M, Aseffa A, Rook G, Fletcher H, Zumla A, Weldingh K, Brock I, Andersen P, Doherty TM (2004). Healthy individuals that control a latent infection with Mycobacterium tuberculosis express high levels of Th1 cytokines and the IL-4 antagonist IL-4delta2. J Immunol.

[B32] Fletcher HA, Owiafe P, Jeffries D, Hill P, Rook GA, Zumla A, Doherty TM, Brookes RH (2004). Increased expression of mRNA encoding interleukin (IL)-4 and its splice variant IL-4delta2 in cells from contacts of Mycobacterium tuberculosis, in the absence of in vitro stimulation. Immunology.

[B33] Leung E, Hong J, Fraser A, Krissansen GW (2006). Splicing of NOD2 (CARD15) RNA transcripts. Mol Immunol.

